# Pulmonary, aorta, and coronary arteries post-arterial switch in transposition of great arteries: intermediate-term surveillance utilizing conventional echocardiography and cardiac multislice computed tomography

**DOI:** 10.1186/s13052-024-01686-x

**Published:** 2024-06-26

**Authors:** Shaimaa Rakha, Nihal M. Batouty, Ahmad AbdelAleem ElDerie, Amira Hussein

**Affiliations:** 1 Pediatric Cardiology Unit, Department of Pediatrics, Faculty of Medicine, Mansoura University, El Gomhouria St, Mansoura, Dakahlia Governorate 35516 Egypt; 2 Department of Diagnostic and Interventional Radiology, Faculty of Medicine, Mansoura University, Mansoura, Egypt; 3 Department of Cardiothoracic Surgery, Faculty of Medicine, Mansoura University, Mansoura, Egypt

**Keywords:** Pulmonary artery, Coronary arteries, Post arterial switch, Transposition of great arteries

## Abstract

**Background:**

Arterial switch operation (ASO) is the standard surgical choice for D-transposition of great arteries (D-TGA). However, the implications of ASO on pulmonaries, coronaries, and aorta have not been adequately investigated. The current study evaluates arterial morphologic changes post-ASO at intermediate-term surveillance.

**Methods:**

From May 2021 to May 2022, patients with D-TGA who underwent ASO for more than six months were recruited. Preoperative and operative data were collected. Patients were assessed using echocardiography (ECHO) and multislice CT angiography (MSCT) to evaluate pulmonary, coronary, and aortic arterial anatomy.

**Results:**

Twenty patients were included with median age of 11 (10-23.25) days at ASO and 14 (7.25–32.75) months on last follow-up. Neo-aortic regurgitation was detected in 12(60%) and neo-pulmonary regurgitation in 3 (15%). Using ECHO, complete evaluation of pulmonary arteries (PAs) was not achieved in 35% and incomplete coronaries assessment in 40% of cases. No stenosis was detected in coronaries using MSCT, although coronary anomalies were found in 9/20 (45%). Dilated Aortic annulus was detected in 16/20 (80%), dilated aortic root in 18/20 (90%), and dilated sinotubular junction in 70%. Right PA stenosis was diagnosed in 10/20 (50%) and left PA(LPA) stenosis in 7/20 (35%). Although Z-score of PAs did not correlate with aortic data, LPA bending angle was positively correlated to neo-aortic root diameter and Z-score (rho = 0.65,*p* = 0.016; rho = 0.69,*p* = 0.01), respectively.

**Conclusion:**

Echocardiography alone is not a conclusive surveillance tool for detecting late post-ASO anatomic changes in D-TGA patients. Cardiac MSCT should be considered for comprehensive evaluation on the intermediate-term follow-up post-ASO to accurately track morphologic abnormalities in the aorta, pulmonary, and coronary arteries.

## Introduction

Dextro-transposition of the great arteries (D‐TGA) is the second most prevalent cyanotic congenital heart disease (CHD). Globally, it accounts for 3.82% of all CHDs, with a prevalence of about 0.3 per 1000 live births [1]. Adib Jatene first performed the arterial switch operation (ASO) in 1975 [2]. In 1981, the Lecompte maneuver was described as a technical modification of surgically translocating the great vessels, avoiding using a prosthetic conduit [3]. In combination with the Lecompte maneuver, ASO has become the ultimate surgical choice for infants with D‐TGA. The ASO allows the morphological left ventricle to remain the systemic ventricle and avoids multiple atrial incisions and suture lines that predispose to arrhythmias as encountered in atrial switch [4].

Transitioning from the atrial switch procedure to ASO for D-TGA has decreased early mortality and morbidity with increased long-term survival [5–8]. However, a long list of reported late complications of the operation substantially affects coronary arteries, neo-aorta, and neo-pulmonary arteries [9–11]. Therefore, many patients who undergo the ASO eventually require reintervention or reoperation [12–14].

Previous studies have reported variability in the onset of development or course of arterial complications progression after the ASO [9, 15, 16]. The concerns regarding the arteries’ fate, neo-pulmonary, and neo-aortic valvular functions warrant persistent surveillance of these patients. Hence, regular follow-up echocardiography (ECHO) post-ASO is currently the standard surveillance tool in most centers [17]. Nevertheless, ECHO usually has limitations in comprehensively evaluating cardiac structures in patients post-ASO [18, 19].

The study aims to evaluate patients with D-TGA following ASO at the intermediate term to document the structural changes and complications of the neo-pulmonary artery and its branches, coronaries, and neo-aorta using ECHO and cardiac multislice CT angiography (MSCT).

## Subjects and methods

The study was a single-center observational study. The recruited patients were D-TGA patients who underwent ASO for more than six months at the time of the study. Out of 37 cases that fulfilled the inclusion criteria, 20 patients could be recruited. The research was approved by the IRB (institutional review board) of Mansoura University, faculty of medicine. Informed consent was obtained from the legal guardian of the participating child. Cases with significant arrhythmia that might impact ECG gating for coronary artery assessment were excluded from the study.

### Preoperative and operative data

Initial preoperative data collected included gestational age at birth, prenatal/postnatal diagnosis, associated cardiac lesions, and the need for balloon atrial septostomy. Operative data included age and weight at ASO and additional cardiac operations required with ASO.

### Demographics on the last follow-up

The patient’s age, weight, and height were documented on the last follow-up visit.

### Echocardiophy (ECHO)

On the last follow-up, conventional two-dimensional, color flow, and pulsed/continuous Doppler ECHO were performed. ECHO.

was used to assess the neo-aortic valve, including the presence of aortic regurgitation and its degree; coronaries were assessed regarding anatomic anomaly or apparent stenosis; and the neo-pulmonary valve for stenosis or regurgitation. Pulmonary artery (PA) branches were evaluated for stenosis with pressure gradient measurement. In addition, systolic myocardial function was assessed using M.mode to measure fractional shortening in percentage.

### Cardiac multislice CT angiography (MSCT)

**Fig. 1:**
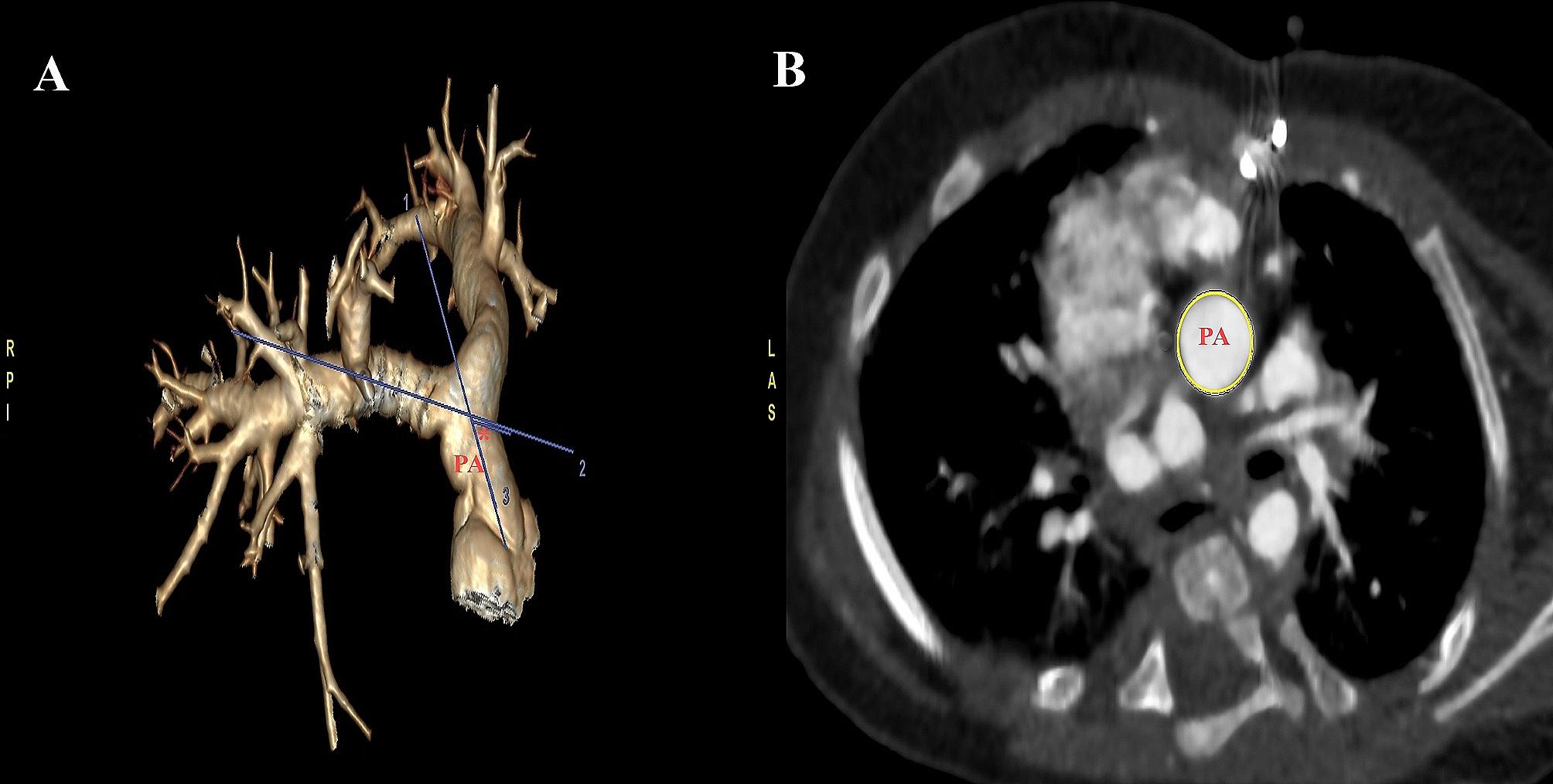
**(A)** Volume-rendered CT images of the pulmonary arteries showing the pulmonary artery bending angle (red asterisk) as the outer angle between main pulmonary artery axis (axis 1) and branch pulmonary artery axis (axis 2), **(B)** Axial oblique CT image showing the cross-sectional area of the pulmonary artery (yellow circle). PA: main pulmonary artery

Patients were recruited for low-dose electrocardiogram-gated 128-slice CT angiography (80 kVp, 150–200 mA, collimation 128 × 0.6 mm) on the last follow-up visit. Contrast medium (concentration: 350 mg iodine/mL) was injected intravenously (dose = 0.5–2 mL/kg, rate = 1–2 mL/s). The scan extended from the origin of aortic arch branches at the lower neck to the upper abdomen. Propranolol 1 mg/kg/day was given 24 h before the CT. The MSCT was performed after conscious sedation of the patients. The cardiac structures’ diameter was indexed as Z-score to body surface area if possible, according to Lopez et al. data [20]. A Z-score of a cardiac structure of more than + 2 was considered dilated, and a Z-score less than − 2 was defined as stenosis. The measurements included the following parameters:

1. For the neo-pulmonary arteries, PAs were reformatted in multiple planes, and the following measurements were taken: main PA diameter, proximal diameter of right and left pulmonary artery (RPA, LPA), narrowest PAs diameter, cross-sectional area of PAs, and pre-branching diameter. The main PA axial angle was calculated as the angle between neo-pulmonary and neo-aortic roots from the median plane [21]. Cases with positive angle are defined as the angle with the main PA rightward from the median plane, and negative angle is for the angle when main PA is leftward of the median plane, and zero angle with direct anterposterior relation with no angle detected between main PA and neoaorta. The PA bending angle was measured as the outer angle between the main PA and branch PA axes [21]. (See Fig. 1)

2. For the neo-aorta, the thoracic aorta was reformatted in multiple planes. The following measurements were taken: neo-aortic root (Sinus of Valsalva) diameter and area, sinotubular junction (STJ) diameter and area, ascending aorta (at the level of PA bifurcation) diameter and area, the diameter of the aortic annulus, proximal arch, distal arch, isthmus, and descending aorta at the level of the diaphragm. Additionally, abnormal anatomic findings were reported.
3. For coronaries, anatomic assessments were performed to document abnormality in branching, position, presence of kinking, stenosis, or occlusion. Diameters and Z-scores were measured for the main left and right coronaries, left circumflex, and left anterior descending. Coronary angle and Coronary-pulmonary bifurcation distance were calculated as described by Ou et al. [22].

### Statistical analysis

The statistical analyses were conducted using appropriate statistical tests on SPSS software (version 25, IBM Corp., Armonk, NY, USA). The normality of variables was tested using the Shapiro–Wilk test. The results for the continuous variables were presented as mean ± SD while non-normally distributed data were presented as median (interquartile range) and the categorical variables as frequencies and percentages. For variable correlations, the Spearman coefficient was used. Logistic regression analysis was performed to determine the effect of variables on the presence of RPA and LPA stenosis. The *p*-value was considered significant when less than 0.05.

## Results

From May 2021 to May 2022, data from 37 cases were initially retrieved from cardiac surgical records as they fulfilled the inclusion criteria. However, only 20 (54.05%) patients could be recruited for the study, 9 (24.32%) patients were excluded due to guardian refusal to consent for participation, 2 (5.41%) were reported by family members as dead due to sudden cardiac death, and 6 (16.22%) patients lost to follow-up.

Table 1 shows the demographic and clinical data of the included cases. Median age at the ASO was 11 (10-23.25) days. Most cases were within the neonatal period except for three cases (15%); two patients had a significant VSD, and one was post-PA banding. The most common associated cardiac anomaly was VSD in 40% of patients, followed by coronary anomalies in 30% of patients. In 40% of patients, an additional surgical maneuver was required with ASO, such as VSD, coarctation repair, neo-aortic root reduction, and PA debanding. The median age at the last follow-up visit was 14 (7.25–32.75) months.

**Table 1:** Patients’ characteristics

Parameter		Data*
Preoperative	**Gestational age **(weeks)	38 (37-39.5)
	**Prenatal diagnosis**	2 (10%)
	**Gender** (Male)	11 (55%)
	**Preoperative Associated anomalies**:	
	• VSD • Coarctation of the aorta • Coronary artery anomalies • Bicuspid aortic valve • Pulmonary stenosis	8(40%) 1(5%) 6(30%) 3(15%) 2(10%)
	**Balloon atrial septostomy**	7(35%)
Operative	**Age at ASO** (days)	11 (10-23.25)
	**BW at ASO** (kg)	3.35 (2.85–3.93)
	Operation performed**	
	• ASO • ASO + VSD repair • ASO + VSD repair + COA repair • ASO + VSD repair + neoaortic root reduction • ASO + neoaortic root reduction+ PA debanding	12(60%) 4(20%) 1(5%) 2(10%) 1(5%)
Last Follow-up	**Age at last Follow-up** (months)	14 (7.25–32.75)
	**BW at last follow-up (kg**)	10.37 ± 2.728
	**Ht at last follow-up** (cm)	81.45 ± 15.77
	**BMI at last follow-up**	15.92 ± 3.27

**ASO**: arterial switch operation, **BSA**: body surface area, **BW**: body weight, **BMI**: body mass index, **Ht** : height.

* Data are presented as mean ± SD, or median (interquartile range),or frequency (Percentage)

**All cases required ASD/PFO ± PDA closure during surgery

**Fig. 2:**
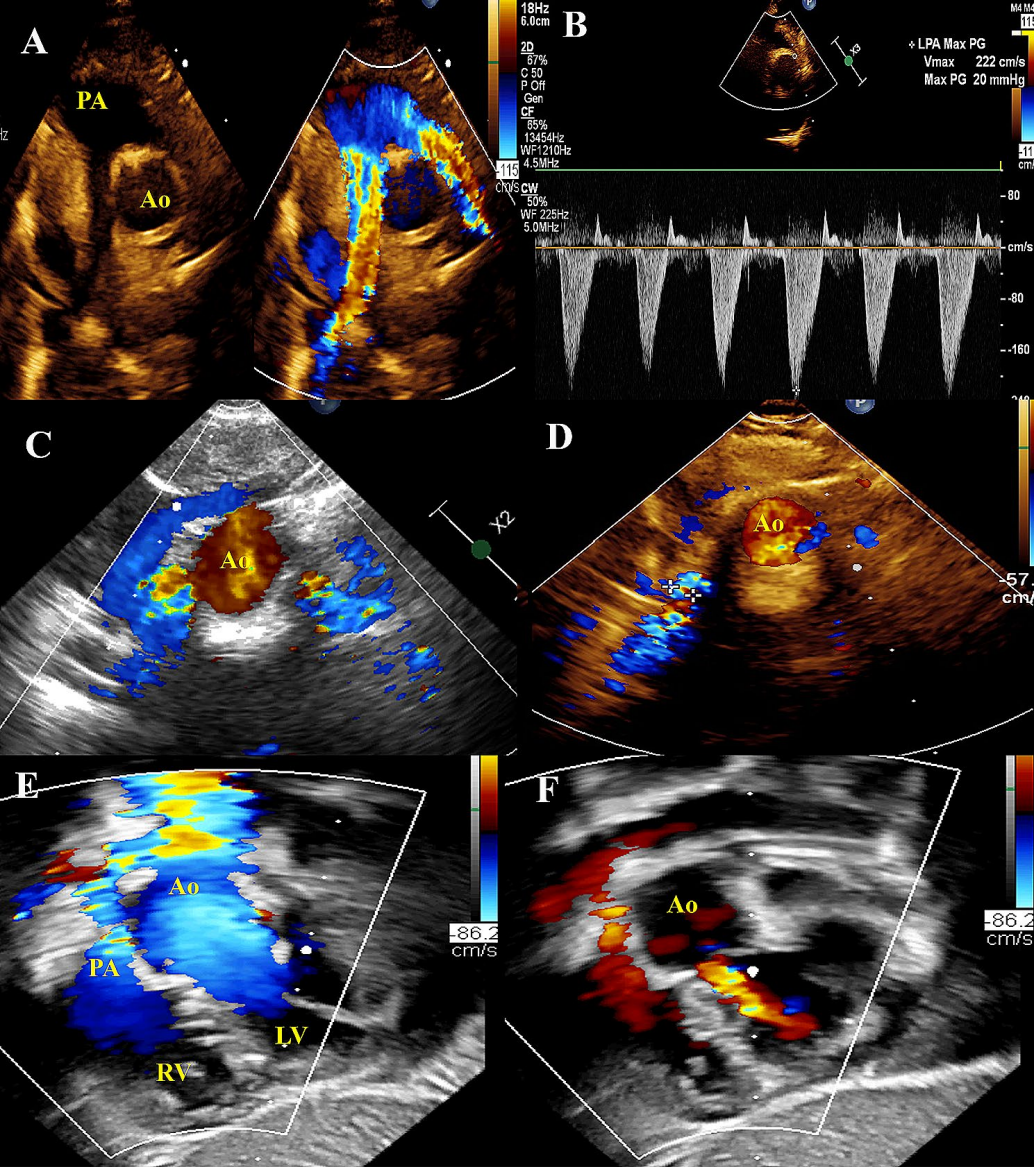
Echocardiographic images of some of the study cases. **(A)** Both LPA and RPA branches are clearly seen draping around the neo-aorta in suprasternal view in the first patient. **(B)** Continuous wave Doppler showing pressure gradient of 20mmhg across LPA in the same first patient. **(C)** Distal parts of both PAs are seen with no visualization of the bifurcation and proximal parts in suprasternal view in the second patient. **(D)** Third case with only the lower part of RPA is seen as small turbulent branch with LPA is non-visualized in the suprasternal view, although both are described as normal in MSCT. **(E, F)** Subcostal view from a fourth patient showing a dilated aortic root compressing the main PA anteriorly with aortic and pulmonary regurgitation is seen in diastole. Ao: neo-aorta, PA: neo-main pulmonary artery, RV: right ventricle, LV: left ventricle

Table 2 details the echocardiographic data of the included cases on the last follow-up. Neo-aortic valve regurgitation was detected in 12 (60%) patients, mainly trivial to mild in degree, except one had moderate regurgitation. Neo-pulmonary regurgitation was found in 3 (15%), trivial to mild in degree. New aortic coarctation was detected in 2 patients (10%). It was difficult to thoroughly assess at least one of the branch pulmonary arteries in 7 (35%) and at least one of the coronary arteries in 9 (45%). Figure 2 demonstrates ECHO images of some of the study cases.

**Table 2:** Echocardiographic data of included cases in the last follow-up

Parameter		Data*
Aortic	PG across neo-aortic valve, (mm Hg)	6.8(4.22–8.83)
	Neo-aortic Regurgitation, n (%) • Trivial • Mild • Moderate	12/20 (60%) 6/12 (50%) 5/12 (41.67%) 1/12 (8.33%)
	Aortic coarctation	2/20 (10%)
	Supravalvular neo-aortic stenosis	1/20 (5%)
Pulmonary	PG across neo-pulmonary valve, (mm Hg)	10.7 ± 3.2
	Neo-pulmonary Regurgitation (trivial-mild), n(%)	3/20 (15%)
	PG across RPA, mm Hg (*n* = 13)	12 (7-29.5)
	PG across LPA, mm Hg (*n* = 13)	12 (7–22)
	Incomplete visualization of at least one PA branch	7/20 (35%)
Coronary	Incomplete visualization of at least one coronary artery	9/45 (40%)
LV FS%		33.5 ± 8.03
Others	• Parachute mitral valve with moderate MR • Bicuspid aortic valve	1/20 (5%) 3/20 (15%)

**FS**: fractional shortening, **MR**: Mitral Regurgitation, **PG**: pressure gradient

* Data are presented as mean ± SD, or median (interquartile range), or frequency (Percentage)

Table 3 demonstrates post-ASO global cardiac CT-derived pulmonary, coronaries, and aortic data. The mean Z-score of the narrowest RPA and LPA diameters was − 1.84 ± 1.56 and − 1.42 ± 1.8, respectively. However, the median Z-score exceeded + 2 for aortic annulus + 4.5 (+ 2.2 - +6.3), aortic root + 4.07(+ 2.91- +6.09), and STJ + 3.18(+ 0.72 - +5.45). All coronary arteries’ diameters Z scores were within the average range.

**Table 3:** Post-ASO global cardiac CT-derived data for pulmonaries, coronaries and aorta

Parameter		Diameter/ area/angle*	Z score*
Pulmonary	Main PA Diameter (mm)	9.95 (9.05–13.67)	-1.16 (-2.15–0.17)
	Narrowest RPA Diameter (mm)	5.14 ± 1.99	-1.84 ± 1.56
	Narrowest LPA Diameter (mm)	5.89 ± 2.36	-1.42 ± 1.8
	RPA area (mm^2^/m^2^)	77.92 (38.56–109.6)	
	LPA area (mm^2^/m^2^)	62.36 (38.73–96.35)	
	Main PA axial Angle (degree)	0 (0-10.35)	
	RPA bending Angle (degree)	101.27 ± 24.99	
	LPA bending Angle (degree)	95.82 ± 21.99	
Aorta	Aortic annulus Diameter (mm)	14.52 ± 3.13	4.5 (2.2–6.3)
	SOV Diameter (mm)	19.84 ± 3.11	4.07(2.91–6.09)
	SOV area (mm^2^/m^2^)	669.16 ± 151.51	
	STJ Diameter (mm)	15.17 ± 3.88	3.18(0.72–5.45)
	STJ area(mm^2^/m^2^)	516.26 ± 186.11	
	Ascending aorta Diameter (mm)	12.14 ± 2.16	0.34(-0.89–0.63)
	Ascending aorta area (mm^2^/m^2^)	297.07 ± 69.41	
	Isthmus Diameter (mm)	7.82 ± 1.99	-0.65 (-1.58–0.4)
Coronaries	RCA diameter (mm)	1.62 ± 0.32	-0.19 (-0.98-0.63)
	LCA diameter (mm)	1.9 ± 0.34	-0.66(-0.9 - -1.2)
	LAD diameter (mm)	1.51 ± 0.2	0.48 (0.12–0.73)
	LCX diameter (mm)	1.33 ± 0.23	
	RCA-pulmonary bifurcation distance (mm)	6.6 (4.8-9)	
	LCA-pulmonary bifurcation distance (mm)	4.8 (4.1–6.2)	
	RCA angle (degree)	39.09 ± 8.63	
	LCA angle (degree)	47.89 ± 17.79	

**LAD**: left anterior descending artery, **LCX**: left circumflex artery, **LCA**: main left coronary artery, **LPA**: left pulmonary artery, **RCA**: main right coronary artery, **RPA**: right pulmonary artery, **SOV**: Sinus of Valsalva, **STJ**: Sinotubular junction

* Data are presented as mean ± SD, or Median (interquartile range)

**Fig. 3:**
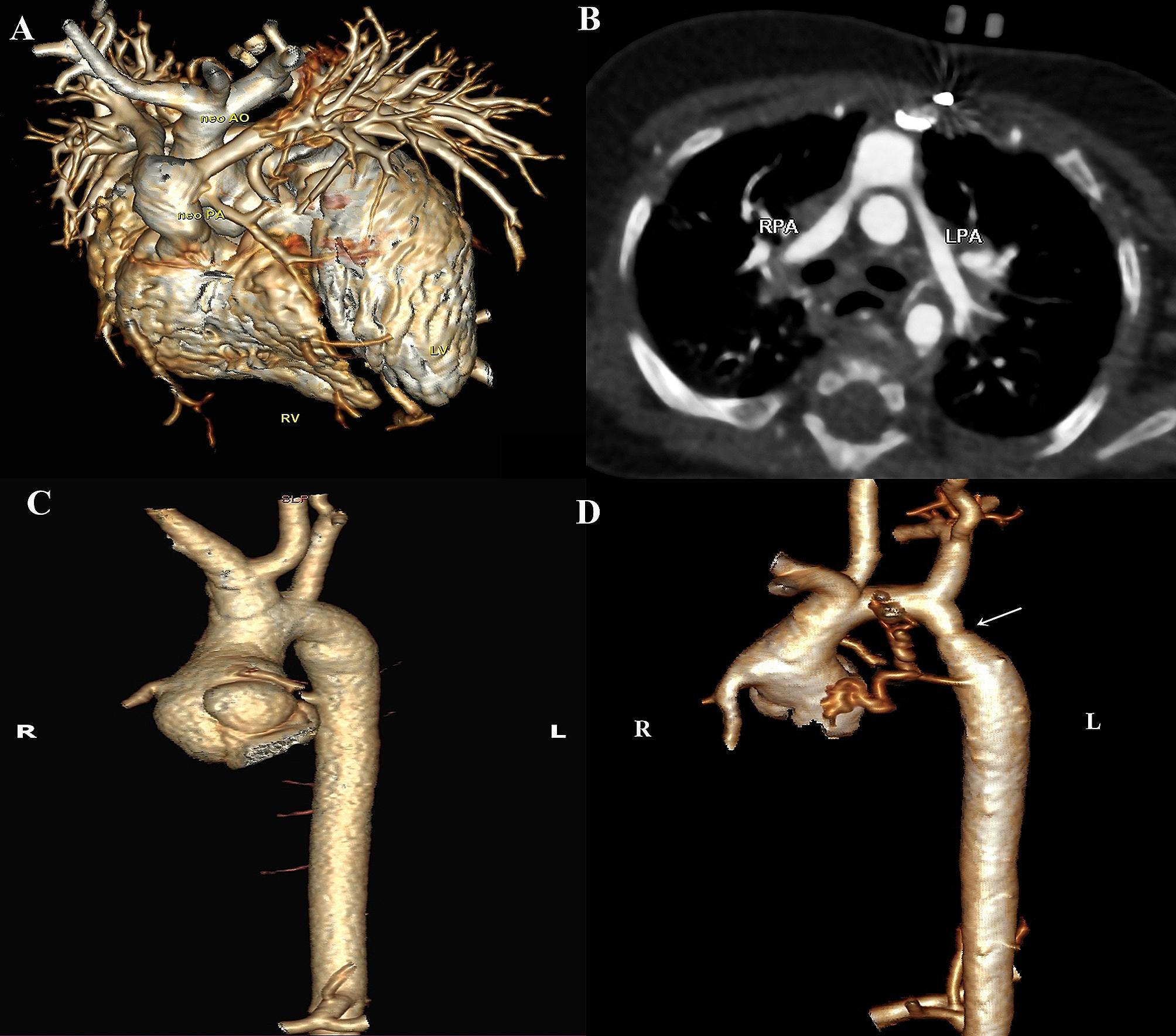
Abnormalities detected in post-ASO MSCT of some of the included cases. **(A)** Volume-rendered CT image showing neo-PA with its two branches draping around the neo-aorta. **(B)** Axial oblique CT image showing stenosis of both RPA and LPA. **(C)** Volume-rendered CT image of the aorta showing dilated neo-aortic root and STJ. Note the common origin of brachiocephalic and left common carotid arteries (Brachiocephalic trunk). high takeoff of both coronary arteries. **(D)** Volume-rendered CT images of the heart and aorta showing aortic coarctation and major aortopulmonary collateral arteries; note the high takeoff of both coronary arteries. LPA: left pulmonary artery, neo-PA: neo-pulmonary artery, neo-Ao: neo-aorta, RPA: right pulmonary artery

The abnormal findings detected in MSCT of the study subjects are summarized in Table 4. Stenosis of at least one of the PA branches was detected in 65% of patients, with RPA stenosis in 50% and LPA stenosis in 35%. Most detected narrowings were in the proximal part of PAs in 80% of RPA stenosis and 85.7% of LPA stenosis. Dilated aortic annulus was predominant in most cases, with 80% of cases having a Z-score more than + 2. Similarly, dilated aortic root was found in 90% of cases and dilated STJ in 70%of study patients. Major aortopulmonary collateral arteries (MAPCAs) were detected in 2 patients (10%) using MSCT but were not visualized on ECHO. Coronary abnormalities were detected in 9 (45%) patients, of which 6 cases were reported at the initial diagnosis or intraoperative, while 3 (15%) were reported with high origin only on CT with no detected coronary arterial stenosis or kinks. Figure 3 illustrates some of the abnormalities detected in post-ASO MSCT.

**Table 4:** Abnormal findings detected in the Cardiac MSCT of the included cases

Parameter		*N* (%)*	Note
Pulmonary	Main PA stenosis	7/20 (30%)	-2.53 ± 0.5**
	Branch PA stenosis	13/20 (65%)	
	• Bilateral PAs stenosis	4/13	
	• Unilateral PA stenosis Unilateral RPA stenosis Unilateral LPA stenosis	9/13 6/9 3/9	
	RPA stenosis	10/20 (50%)	-3.19 ± 0.83** 2 required intervention
	• Proximal	8/10 (80%)	
	• Pre-branching	1/10 (10%)	
	• Mid	1/10 (10%)	
	LPA stenosis	7/20(35%)	-2.93 ± 0.57** None required intervention
	• Proximal	6/7 (85.71%)	
	• Pre-branching	1/7 (14.29%)	
Aorta	Dilated Aortic annulus	16/20 (80%)	
	Dilated Aortic Root	18/20 (90%)	
	Dilated STJ	14/20(70%)	
	Dilated Ascending aorta	1/20 (5%)	
	Supravalvular aortic stenosis	1/20 (5%)	1 required intervention
	Aortic coarctation	3/20 (15%)	1 required intervention
	Aortic arch abnormalities		
	• Gothic arch	1/20 (5%)	
	• Crenel arch	1/20(5%)	
	• Rt aortic arch	1/20(5%)	
	• Aberrant RSCA	1/20(5%)	
Coronaries	Coronary abnormality:	9/20 (45%)	
	• All coronaries from common origin (Yacoub Type B)	3/20 (15%)	
	• common origin of RCA + LCX from Rt sinus and LAD from anterior STJ (Yacoub Type D)	2/20 (10%)	
	• High Origin of one or both Coronaries above STJ	3/20 (15%)	
	• LCA arises from Rt sinus, LAD retroaortic	1/20(5%)	
Others	MAPCAs	2/20 (10%)	

LAD: left anterior descending artery, LCX: left circumflex artery, LCA: main left coronary artery, LPA: left pulmonary artery, MAPCAs: Major aortopulmonary collateral arteries, RCA: main right coronary artery, RPA: right pulmonary artery, STJ: Sinotubular junction

* Data are presented as frequency (Percentage), ** Mean ±SD of PA diameter in cases with stenosis

No significant linear correlations were detected between aortic-related diameters and PA branches diameters, Z-scores, areas, or pressure gradients. Logistic regression analysis was performed to determine the effect of branch PA bending angle, aortic root, and STJ Z-scores on the presence of RPA and LPA stenosis; none yielded a significant result. On investigating the linear correlation of the PA branches bending angle with other aortic-related measurements in cases with PA stenosis (Table 5), LPA bending angle was positively correlated to neo-aortic root diameter and Z-score (rho = 0.65,*p* = 0.016; rho = 0.69,*p* = 0.01), respectively and with aortic isthmus diameter and Z-score (rho = 0.54,*p* = 0.048; rho = 0.64,*p* = 0.02), respectively. Moreover, the RPA bending angle was negatively correlated with cross-sectional area and Z-score, indicating that increasing the bending angle was associated with a decrease in the Z-score of the RPA; a similar nonsignificant relation was detected with LPA.

**Table 5:** Linear correlation of PA branches bending angle with aortic-related measurements and cross-sectional area and Z score of PA in cases with PAs stenosis

	RPA Bending Angle		LPA Bending Angle	
	***rho***	***P***	***rho***	***P***
Aortic annulus*	-0.48	0.1	0.21	0.49
Aortic annulus Z	-0.28	0.36	0.11	0.72
SOV*	-0.19	0.55	0.65	0.016**
SOV Z	-0.05	0.87	0.69	0.01**
SOV area/SA	-0.41	0.23	0.75	0.04**
STJ*	0.13	0.67	0.11	0.71
STJ Z	0.16	0.6	-0.09	0.75
Ascending Aorta*	-0.11	0.71	0.36	0.23
Ascending Aorta Z	0.24	0.43	0.53	0.06
Isthmus*	0.09	0.77	0.54	0.048**
Isthmus Z	0.31	0.31	0.64	0.02**
MPA axial angel	-0.41	0.16	0.31	0.3
Cross-sectional area of the respective PA	-0.78	0.008**	-0.41	0.20
Z-score of the respective PA	-0.517	0.13	-0.43	0.34

LPA: left pulmonary artery, MPA: main pulmonary artery, RPA: right pulmonary artery, SOV: sinus of Valsalva, STJ: Sinotubular junction, p: Significance value (2-tailed), rho: Spearman coefficient, Z: Z-score

* Diameter

**p value significant if ≤ 0.05

## Discussion

ECHO is a mandatory surveillance tool post-ASO for determining the presence of regurgitation in the neo-aorta or the neo-pulmonary valve and for estimating pressure gradient across valves, neo-PAs, or neo-aorta. Nevertheless, several limitations are encountered when using ECHO alone for mid-term surveillance, especially for evaluating neo-PAs and coronaries. In the current study, we comprehensively investigated a cohort of patients using ECHO in combination with complementary data retrieved from cardiac MSCT angiography, which was more detailed and accurate for intermediate-term follow-up.

It was technically limited to thoroughly evaluate at least one PA using ECHO in 35% of cases. However, Morgan et al. reported the limitation of ECHO in evaluating PAs post-ASO in 21% of cases, as they found it was technically not visible to perform PA Doppler [19]. In another study, an insufficient acoustic window for PA evaluation was the reason for the referral of all cases post-ASO for cardiac magnetic resonance (CMR) [18].

In some cases, it is challenging to visualize the Lecompte maneuver-resulted PAs bifurcation, as it could be highly situated in the neck that we could not visualize or for the limited acoustic window. In other cases, we could not accurately visualize the anatomy of the neo-PA stenosis despite apparent turbulence on color flow or turbulence that could be a prominent flow without a true stenosis. Sometimes, it is difficult to visualize the PA conclusively by ECHO, or the degree of obstruction could be underestimated because severity estimates rely on Doppler velocities, dependent on volumetric flow which is redistributed to the healthier lung, yielding low gradients [23]. Even severe unilateral PA obstruction often minimally affects right ventricular systolic pressures. Furthermore, the physiologic limitations of PA stenosis could remain insidious, eventually resulting in decreased exercise capacity and limited activity [24]. However, lung perfusion and exercise capacity improve after percutaneous treatment for branch PA stenosis [25]. Moreover, in some of our patients, the bending angle of PA could make it challenging to align the cursor properly for accurate pressure gradient estimation.

Stenosis of at least one of the neo-PA branches was confirmed using MSCT in 65% of the patients in the current series, of which two required intervention. The most commonly affected was RPA in 50% of the patients. On the contrary, Loke et al. found that LPA stenosis was more common (in 78% of cases), while 47% had RPA stenosis. Morgan et al. found that 28% had decreased flow to the left lung. They proved that neo-pulmonary to neo-aortic geometry and post-operative compression of the LPA by the enlarged aorta impact LPA size and perfusion of the left lung. Aortic root diameter correlated inversely with LPA flow but not with LPA cross-sectional area [19]. Similarly, in our series, aortic root diameter and other neo-aorta-related parameters did not correlate to the cross-sectional area or Z-score of PAs.

It was suggested that the systolic expansion of the neo-aorta compresses the main neo-PA or the branches. Moreover, the decreased PA branch diameters could be due to the branches stretching over the dilated neo-aorta [26]. Lecompte maneuver may involve stretching the PA branches while moving the bifurcation anterior to the proximal neo-ascending aorta. The anastomosis to the neo-main PA may be under tension despite patch arterioplasty, and PA branches could also be compressed as they are splayed around the ascending aorta with the classical technique for PA reconstruction, the single pantaloon pericardial patch technique [27, 28]. The neo-PA or branch PAs may be near the coronary artery buttons implanted into the neo-ascending aorta [29].

Regarding the bending angle of the PAs, the RPA bending angle was slightly wider than the LPA angle. A similar finding was reported by Loke et al., but they detected a smaller angle means for both RPA and LPA than in our series. They concluded that the abnormality in PA angle bends following ASO correlates with increased RV afterload regardless of PA stenosis [21]. However, in our studied cohort, the RPA bending angle of the cases with RPA stenosis was negatively correlated with the cross-sectional area and Z-score of RPA, indicating that increasing the bending angle was associated with a decrease in the Z-score of the RPA; a similar nonsignificant relation was detected with LPA.

Furthermore, using CMR with computational fluid dynamics, Capuano et al. proved that there are anomalous shear layer instabilities, vortical and helical structures, and turbulent states in patients post-ASO, particularly as a sequel of the unnatural curvature of the PA bifurcation and that the geometrical features promoted the separation of the flow at the PA branch entrance in post-ASO patients, recirculation areas, and subsequent flow instability. They found the RPA bending angle was significantly smaller (more acute) than the control’s, while the LPA angles were similar. However, they studied only 3 cases and one control [30].

On measuring the main PA axial angle, 70% of our cohort presented a direct anteroposterior great arterial arrangement, with 25% showing a rightward neo-pulmonary root. This could partly explain the small prevalence of LPA stenosis in our series. On the contrary, Morgan et al.‘s series included 83% rightward neo-pulmonary and only 5% direct anteroposterior arrangement with more frequency of LPA stenosis in their cohort [19].

Aortic root dilation was detected in 90% of the current series, aortic annulus dilatation in 80%, and STJ dilatation in 70%. Van der Palen et al. found that after a rapid increase in the first year post-ASO and proportional growth in childhood, neo-aortic dimensions continue to increase in adulthood for neo-aortic annulus, root, and STJ, all significantly exceeding average growth [9]. Aortic root dilation is a recognized complication post-ASO and is generally well-tolerated [31]. Risk factors for developing aortic root dilation include prior pulmonary arterial banding, Taussig-Bing anatomy, and the presence of a VSD [32]. Aortic root dilation could result from maladaptation of the former PA (the neo-aorta post-ASO) to the higher systemic pressure it is exposed to post-operative or could be due to scarring around surgical suture lines, which reduces root elasticity and distensibility [33, 34].

Despite establishing ventriculoarterial concordance after ASO, abnormal flow patterns confirm that the usual spiral configuration of the great vessel arising from the heart was not restored [35]. These abnormal flow patterns with increased flow helicity result in increased shear stress, which may contribute to root dilation. In computational modeling, the spiraling of the great vessels results in a more physiological passage of blood from the ventricles [36]. Therefore, the spiraling arrangement of great arteries prevents the compression of PAs by the aorta due to the wide window in the new aortic arch and reduces stretching and compression of the branch PAs [37, 38]. Moreover, the aortic root dilatation was found to be a risk factor for neo-aortic regurgitation [32]. In our series, all cases of neo-aortic regurgitation have aortic root dilation. Koolbergen et al. found that reoperation for root dilatation or aortic regurgitation could be required unrelated to a specific risk factor [39]. Neo-aortic valve regurgitation in the current series (60%) was significantly higher than other recent reports, as mild regurgitation was detected in 3.7% of Nguyen et al. cohort, while Wang et al. reported regurgitation in 11.45% of their patients [40, 41]. Although infrequently reported, supravalvular neo-aortic stenosis could develop at the reconstructed STJ requiring reoperation [42–44]. We had one case that developed early postoperative and progressed to severe pressure gradient by age of 7 months when he was referred to repair.

Unusual coronary artery anatomy was detected in 45% of our cohort. A higher prevalence of detected abnormal anatomy post-ASO was detected by Morgan et al. in 67% of patients [19]. Transfer of the coronary artery origins is critical to a successful ASO. Coronary artery reimplantation to the neo-aorta has been performed using different techniques, including various shaped button and trap door techniques [45]. The split incision for coronary reimplantation technique can alleviate coronary artery tension and avoid coronary artery distortion or obstruction. At the same time, it also enlarges the neo-aortic root and increases the risk of aortic regurgitation [46]. In the current study, no cases had stenosis or kinking at any level of the coronaries. Ou et al. reported significant coronary stenosis in 8.9% of their series detected using MSCT, but in a cohort of older childhood age (5–16 years) [22]. Furthermore, Linglart et al. described a significant prevalence of myocardial ischemia post-ASO in their series, reaching 27.5% of patients with coronary-related mortality in 10% of the symptomatic patients [47].

In most cases, D-TGA patients underwent ASO without doing an initial CT; hence, several arterial abnormalities were not documented earlier, and others developed later. Some abnormalities in the shape of the aortic arch were not documented before the operation, such as a case with a crenel arch and another with a gothic arch. In the current report, MAPCAs were accidentally detected on MSCT of two cases but with no hemodynamic impact. MAPCAs from the descending aorta to the pulmonary arteries are uncommon in D-TGA, especially after ASO. The reported prevalence of MAPCAs in D-TGA was 1.9%, with about two-thirds after ASO [48]. Rarely can they cause critical deterioration, pulmonary bleeding, or heart failure, necessitating closure through coil embolization or vascular plug [49–51].

The major limitation of the current work is the single-center design with a small sample size. A more extensive multicenter study is required to prove the correlations between aortic root, PA size, and bending angles. One of the drawbacks of using CT is always the radiation, and to avoid the radiation, CMR could be preferred. Although few studies reported post-ASO evaluation using CMR, the coronary assessment was not performed [18, 26]. Unlike CMR, CT is not hindered by metallic devices. CT with three-dimensional postprocessing techniques, a wider detector system, ECG-dependent modulation, and dose-reduction strategies are valuable in assessing post-ASO complications [52].

## Conclusion

Echocardiography alone is not a conclusive surveillance tool for detecting post-ASO anatomic changes in D-TGA patients. Cardiac MSCT should be considered for comprehensive evaluation on intermediate-term follow-up of children post-ASO to accurately track abnormalities in the neo-aorta, neo-pulmonary, and coronaries. Delineating the mechanisms of obstructive complications after the ASO and understanding these aspects could help improve the surgical technique to minimize the risk of late sequelae.

## Data Availability

The datasets used and/or analyzed during the current study are available from the corresponding author upon reasonable request.

## Abbreviations

ASO: Arterial switch operation
CMR: Cardiac Magnetic Resonance
CT: Computed Tomography
D-TGA: Dextro-transposition of the great arteries
ECG: Electrocardiogram
ECHO: Echocardiography
LPA: Left pulmonary artery
MAPCAs: Major aortopulmonary collateral arteries
MSCT: Multislice CT
PA: Pulmonary artery
RPA: Right pulmonary artery
STJ: Sinotubular Junction
VSD: Ventricular Septal Defect
